# Getting to the Warm Hand-Off: A Study of Home Visitor Referral Activities

**DOI:** 10.1007/s10995-018-2529-7

**Published:** 2018-06-02

**Authors:** Jessica Goldberg, Jessica Greenstone Winestone, Rebecca Fauth, Melissa Colón, Maria Verónica Mingo

**Affiliations:** Tufts University, 574 Boston Ave., Medford, MA 02155 USA

**Keywords:** Home visiting, Service coordination, System of care, Referrals

## Abstract

**Introduction:**

Conducted as part of the Massachusetts MIECHV evaluation, this study examined the role of home visitors (HVs) in facilitating families’ connections to early childhood systems of care. The aims of this study were to document the full range of HV behaviors related to service coordination.

**Methods:**

The study sample was 65 participant cases from five program sites, comprising two home visiting models (HFM and PAT). We coded and analyzed 11,096 home visiting records, focusing on identifying referrals, connections, disconnections, and supportive behaviors across 20 service areas. Qualitative pattern analyses were conducted on a subsample of records to identify unique pathways from referral to connection.

**Results:**

HVs discussed an average of 30 different programs with each participant, and overall, only 21% of referrals resulted in a service connection. This rate varied, with some (e.g., housing) requiring much more intensive HV support and yielding far fewer connections. HVs also worked to keep participants engaged once they were connected to a service, often discovering challenges in need of attention through monitoring activities.

**Discussion:**

Home visiting is often thought of as a key entry point into a system of care. Findings from this study confirm this premise, highlighting both the centrality of home visiting in helping families navigate local systems of care, and the insufficiency of these systems to meet family needs.

## Significance

To date, there have been no comprehensive evaluations of service coordination within the home visiting context, particularly regarding the pathways that lead from referral to connection. This study of referral-making and service coordination in the Massachusetts MIECHV home visiting program, which details home visitors’ efforts to help participants navigate services in their local systems of care, begins to address this gap in the literature and point to directions for further research.

## Introduction

Authorized as part of the 2010 Affordable Care Act, the Maternal, Infant, and Early Childhood Home Visiting program (MIECHV) provides federal funds to states and tribal entities to support evidence-based home visiting services to families in at-risk communities. MIECHV has a stated focus on early childhood systems building (Patient Protection and Affordable Care Act 2010); this recognition that home visiting is not a panacea, but rather an essential component in a larger system of care (Daro 2009), acknowledges the challenges of providing services to populations with multiple needs, situated in insufficiently resourced communities. There has been increasing interest in understanding how home visiting programs connect families to services and strengthen local systems of care (Minkovitz et al. 2016; Roberts et al. 1996). While literature investigating home visiting impacts on child and family outcomes is plentiful, far less attention has been given to the processes and outcomes associated with referral-making in home visiting. Conducted as part of the implementation evaluation of the Massachusetts (MA) MIECHV program this study is a mixed-methods process investigation of home visitors’ (HV) role in facilitating and maintaining families’ connections to other services in the early childhood system of care.

## Background

MA MIECHV delivers home visiting services via four models [Early Head Start (EHS), Healthy Families America (HFA), Healthy Families Massachusetts (HFM), and Parents as Teachers (PAT)], in 17 high-need communities across MA. MA MIECHV aims to help families across myriad domains (e.g., health, positive parenting, child development, economic self-sufficiency), either directly or by connecting families to resources and supports in their communities; the latter is the focus here.

Linkage to community resources has long been a stated component of home visiting programs’ offerings (Duggan et al. 1999; Olds and Kitzman 1993; Roberts et al. 1996), and the 2010 MIECHV legislation, which includes coordination and referrals to needed services as one of its benchmark domains (Adirim and Supplee 2013), brought this aspect of home visiting into sharper focus. Despite this redoubled emphasis, little is known about whether and how home visiting programs are meeting this service coordination need. As an example, of the 20 home visiting models established as evidence-based (EBHV) by the U.S. Department of Health and Human Services (Avellar et al. 2017), only six considered linkages and referrals in their assessment of program outcomes (Anisfeld et al. 2004; Dodge et al. 2014; Jacobs et al. 2015; LeCroy and Krysik 2011; Love et al. 2002; Lowell et al. 2011; Olds et al. 1986; Silovsky et al. 2011).

These evaluations focused on two service coordination outcomes: whether a referral was made, and whether a connection to service occurred. Programs with a designated case manager/service coordinator on the home visiting team demonstrated success connecting families to community services (EHS, Love et al. 2002; Child First; Lowell et al. 2011), as did programs with a stated focus on service coordination as a key service offering (Family Connects, Dodge et al. 2014; SafeCare Augmented; Silovsky et al. 2011). Findings from evaluations of programs without an explicit focus on service coordination are less consistent; while HFA programs in Kentucky (Williams et al. 2017) and Oregon (Green et al. 2017) successfully connected families to community resources, for instance, other state HFAs (e.g., Hawaii, Massachusetts, Arizona) were found to have no effects on service linkage (Duggan et al. 2004; Jacobs et al. 2015; LeCroy and Krysik 2011).

Evaluations of early intervention (EI) programs (not considered EBHV) have examined service coordination in more depth, perhaps because EI is legislatively mandated to offer case management to participants (Peterson 1991). Qualitative investigations of EI parents’ attitudes around referral-making have found that these families expect and value HVs’ help connecting them to services (Able-Boone et al. 1990; Allen 2007; Mahoney et al. 1990; Roberts et al. 1996). In one of the few studies focusing on service coordination in the context of a HV’s workload, Roberts et al. (1996) found that HVs spent 40% of their time integrating services for families.

To our knowledge, there have been no evaluations that comprehensively explore how service coordination operates within home visiting programs, particularly regarding the pathways that lead from referral to connection. This study, which details HVs’ efforts to help young mothers navigate services in their local systems of care, begins to address this gap in the literature. Our operationalization of referral-making was informed by the information and referral (I&R) services field, which suggests that referrals are more than a one-time event, and emphasizes the importance of follow-up support activities as part of the referral-making processes (AIRS 2016; Gutiérrez 1992; Levinson 2002; Long 1973). The present study documented the *full range* of HV behaviors involved in service coordination, including behaviors intended to identify needs, connect participants to services, and maintain their engagement once connected.

## Methods

For this mixed methods study, we employed qualitative methods to code the data and examine pathways from referrals to connections, and conducted quantitative analyses of referrals, HV support behaviors, and service connections.

### Data Source

Our data source was participant case histories drawn from referral, secondary activity, and home visit records contained within home visiting programs’ web-based management information system (MIS). HVs use this system to track background information about participants (e.g., demographics), service delivery (e.g., the content of home visits, referrals, and other services), goal setting and attainment, and child and mother assessments. Referral records included the description of the program to which the family is referred, and the referral outcome. Secondary activity records described any non-visit activities (e.g., dropping off diapers, contacts with other service providers) conducted by the HV with or for the participant. Home visiting records included HVs’ narrative summaries of what was discussed with, or observed about, the participant across multiple service areas (e.g., housing, child care). We defined a “record” as a single note recorded by a HV in any of these three sources.

### Samples

We used a stratified, weighted sample of 65 participant cases from five demographically diverse program sites comprising two home visiting models (HFM and PAT), randomly selecting cases from each site based on program capacity. The records used for this study covered a 4-year period (2012–2016), encompassing participants’ entire duration in the program.

### Coding

As a first step, a team of six coders reviewed all records for the 65 participants in the sample, retaining those containing any mention of community service-related activities. Next, coders organized these codeable records into “service discussions,” or chronologically ordered records within a participant’s case history related to that one specific program or service. For example, a discussion may start on one visit with the participant expressing a desire to attend college, and subsequent visits may include the HV and participant completing applications and making calls to admissions offices. The last record in which the college is mentioned by the HV would be considered the “end” of the discussion.

Finally, we coded each record in the discussions, using a multi-level scheme capturing all stages of HVs’ facilitation of participants’ linkages to community services, including pre-referral activities (e.g., suggesting a service), referrals (i.e., the initial action taken to link a participant to a service), referral follow-up activities (e.g., assistance completing applications), service connection, service disconnection, post-connection activities (e.g., satisfaction check-ins), and post-disconnection activities (e.g., attempts to re-engage). Codes also characterized the primary goal of each discussion [i.e., to connect a participant to a service (“linking mode”), or support an existing connection (“maintaining mode”)]. We identified a hierarchy of HV behavior codes, based on the intensity of time and effort required from HVs in providing each type of support, including low-level support (*check-ins*), moderate support (*encouragement*/*suggestions*/*advice; emotional support*/*cheerleading; information provision*), and advanced support (*instrumental support; interagency case review*) (see Table 1). A detailed coding manual with definitions and examples guided coders. We achieved an interrater reliability rate of 85% based on 20% of the records.

**Table 1:** Coding scheme: home visitor referral follow-up supports

	Terms	Brief definition	Example
Low-level support	Check-ins	Interactions between HVs and participants that are in the service of tracking and monitoring. HV ask, or participants volunteer information, about progress toward connecting to a service, concerns about service connection, and actions they must take to become eligible for enrollment	*Mom used to have Medicaid but it was closed in February. Mom reapplied and the status is pending*
Moderate support	Encouragement, suggestions, and advice	The HV advises or encourages the participant to take an action related to connecting to a service	*Mom said she didn’t get a chance to call the adult GED program and she will. HV encouraged her to do it ASAP*
	Emotional support/Cheer-leading	The HV demonstrates support, gives praise, or provides comfort related to a service that the participant is attempting to connect to	*I praised mom for keeping her cool when the DTA case manager told her she was no longer eligible for benefits—we talked about how hard it was for mom to not blow her top*
	Information provision	The HV provides the participant with information needed to connect with a service or information about how a service works	*Mom is in the process of applying for unemployment and asked me how to do it. I researched material and went over it with her*
Advanced support	Instrumental support	The HV provides hands-on assistance to the participant (e.g., helps complete an application, accompanies participant to appointment) in order to promote service connection	*I help mom fill out the financial application for child support. It took up the whole visit*
	Interagency case review	Parties from one or more programs, including the home visiting program, discuss a participant’s case	*Had meeting with [Child Protection Services] social worker to talk about Mom’s service plan for reunification*

### Analysis

Overall sample statistics are based on data aggregated at the participant level, and statistics for each service area are based on records nested within discussions. For the qualitative component, we used in-depth pattern analyses to create case studies for 20 participants, randomly selected from the larger sample. In this paper we focus largely on the content and frequency of HV behaviors that occurred *after a referral* (rather than the full range of behaviors), and present two case studies that are particularly good illustrations of the nature of HV-participant interactions around service coordination.

### Consent and Approval

Our research team is granted access to the MIS records through our ongoing evaluation contract with the home visiting program; our research group is named in the informed consent participants sign when enrolling in MA MIECHV. This research was approved by the Tufts University Institutional Review Board.

## Results

In this section we report findings related to: the frequency with which HVs document service coordination activities, and the proportion of activities that are referrals; the distribution of referrals across service areas; the types of referral follow-up supports HVs offer; and the pathways from referral to service connection.

### Frequency of Discussions Related to Service Coordination Activities

As seen in Table 2, there was a total of almost 55,000 records across the 65 participants. Of these, around one-fifth (20.2%, *n* = 11,096) contained mention of a community service; approximately 171 records per participant. These codeable records were organized into, on average, approximately 30 discussions about individual programs per participant, almost a third of which included a referral.

**Table 2:** Home visitor records pertaining to community services

	*N*	*M*	SD	Range
Total records	54,959	845.52	496.48	41–1986
Records containing mention of a community service	11,096	170.70	119.24	1–557
Service discussions	1947	29.95	13.99	1–65
Service discussions including a referral	636	9.78	6.92	0–33

### Distribution of Discussions and Referrals across Service Areas

**Fig. 1:**
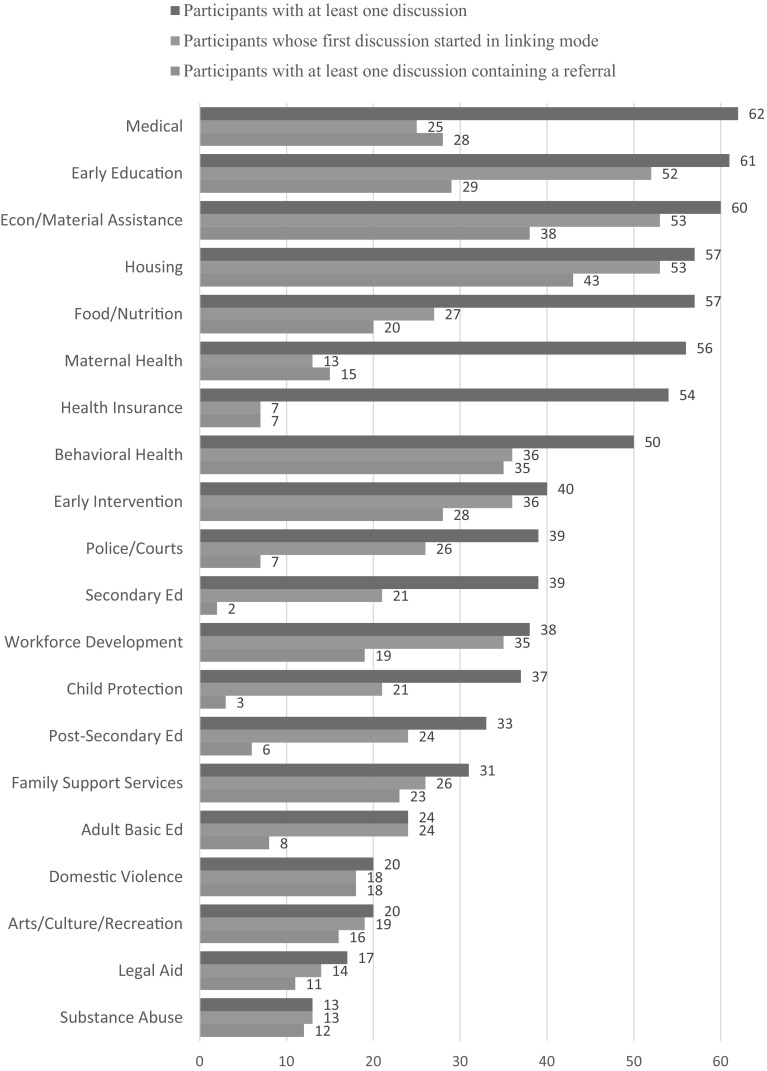
For each service area, proportions of participants who had at least one discussion, had a first discussion beginning in linking mode, and had at least one referral (*n* = 65)

Of the 20 service areas we coded for, participants had discussions pertaining to 13, on average (range 1–20) As shown in Fig. 1, most participants had at least one discussion related to medical (95%), early education and care (94%), economic/material assistance (92%), housing (88%), and food/nutrition services (88%). Even the least common service areas (i.e., substance abuse, legal aid) were featured in at least one-fifth of the participants’ records.

Participants generally had more than one discussion per service area. To understand where participants typically started with respect to each service area (i.e., linking vs. maintaining mode), we examined their first discussions. As shown in Fig. 1, the proportion of initial discussions that began in linking mode varied widely by service area. All of the participants who discussed a substance use program with their HVs, for instance, began these discussions in linking mode (i.e., they were not connected to the service, but likely needed to be), which was true for only a handful of participants who discussed health insurance (i.e., most participants were already connected to health insurance).

Figure 1 also shows the proportion of participants that had at least one discussion that included a referral. In some of the service areas, such as maternal health, behavioral health, domestic violence, and substance use, there was tight congruence between the proportions of participants who started a discussion in linking mode and those who went on to receive at least one referral, suggesting these are areas in which participants were specifically looking for referrals and HVs were well placed to provide them. On the other hand, there was little to no overlap between the two proportions in other service areas, such as child protection, and secondary and post-secondary education, areas in which participants either may not have wanted/needed referrals, or HVs were unable to provide them for some reason (see Fig. 1).

### HV Post-referral Follow-Up Behaviors

For a more granular investigation of HV activities following referrals, we focused on the most prevalent and/or salient service areas in our sample: behavioral health, early education and care, economic/material assistance, food/nutrition, housing, and maternal health. With regard to these service areas, the majority (68%) of HVs’ post-referral activities comprised low-level supports (check-ins), and almost a third (32%) were either moderate or advanced supports; this proportion was similar across service areas.

### Moderate and Advanced Supports

Whereas the overall proportion of low-level to moderate/advanced supports did not differ by service area, the distribution of moderate and advanced supports revealed considerable variation (see Table 3). Referrals to food/nutrition and maternal health services were most often followed by moderate supports, whereas referrals to behavioral health, early education and care, economic/material assistance, and housing services were most often followed by advanced supports.

**Table 3:** Distribution of moderate/advanced supports following home visitor referral, by service area

Service area	Total	Moderate supports						Advanced supports			
		Encouragement, suggestions, advice		Emotional support/cheerleading		Information provision		Instrumental support		Interagency case review	
		*n*	%	*n*	%	*n*	%	*n*	%	*n*	%
Behavioral health	97	21	22	6	6	8	8	23	24	**39**	**40**
Early education	62	16	26	3	5	12	19	**23**	**37**	8	13
Econ/material assistance	31	6	19	0	0	2	6	**23**	**74**	0	0
Food/nutrition	16	**9**	**56**	0	0	4	25	3	19	0	0
Housing	77	11	14	1	1	21	27	**42**	**55**	2	3
Maternal health	12	**6**	**50**	1	8	1	8	2	17	2	17

Bold type indicates the most common type of moderate/advanced home visitor support following referrals in each service area

### Proportion of Referrals Ending in Connection

Overall, 21% of referrals resulted in a connection to the service, with referrals to behavioral health, economic/material assistance, food/nutrition, and maternal health service proving more successful than referrals to child care and housing (see Table 4).

**Table 4:** Referrals and connections, by service area

Service area	Total referrals	Connections to service	
		*n*	% of Referrals
Behavioral health	65	18	28
Early education and care	67	7	10
Economic and material assistance	75	20	27
Food and nutrition	33	10	30
Housing	88	11	13
Maternal health	21	6	29
Total	349	72	21

### HV Support Behaviors between Referrals and Connections

**Fig. 2:**
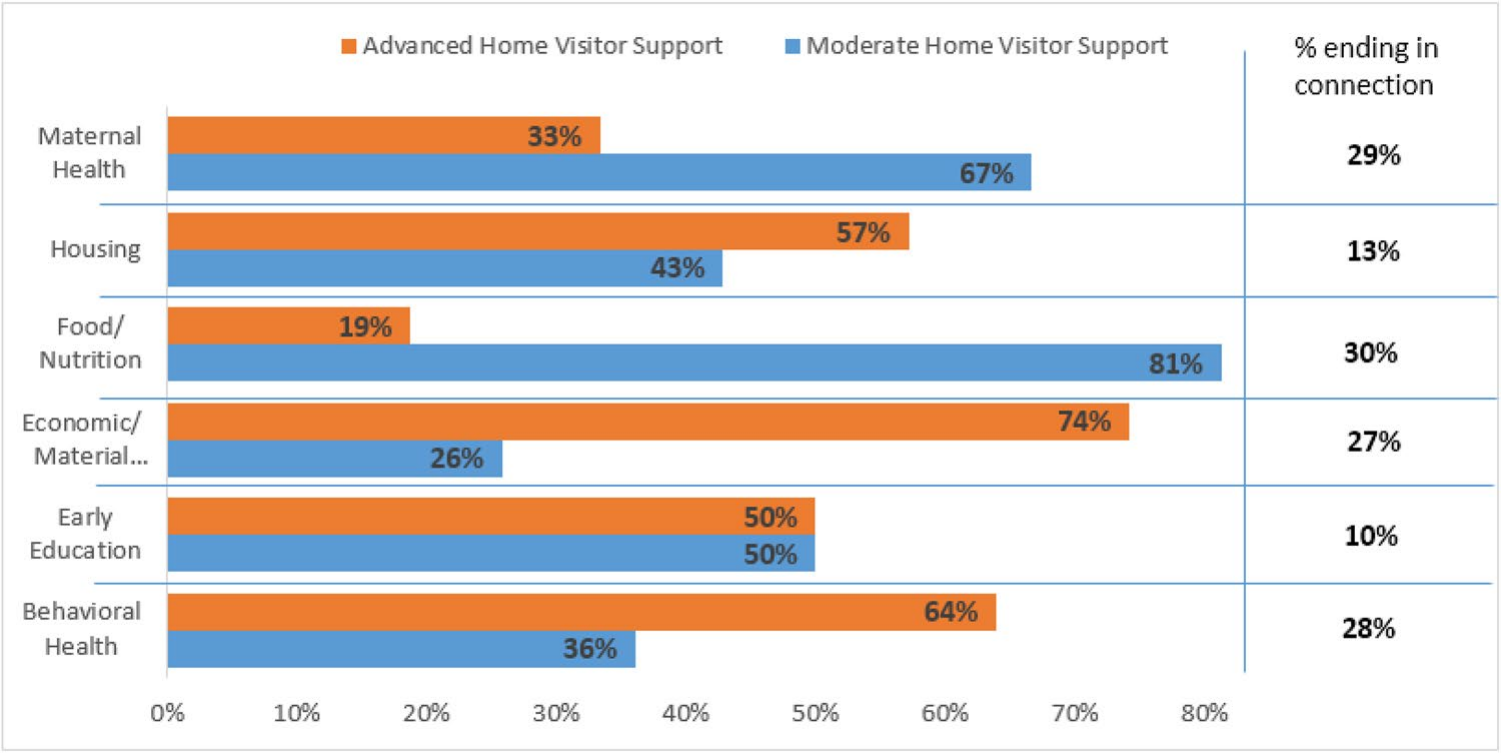
Distribution of moderate and advanced supports preceding connection to services, by service area

Figure 2 shows the intensity of HV effort (moderate or advanced support) relative to the success in connecting for each of the six service areas. Referrals to maternal health and food/nutrition services required only moderate effort, and resulted in a relatively high number of connections. Referrals to behavioral health services and economic/material assistance required more intensive follow-up, but yielded similar rates of connection. Early education and housing also required more advanced effort, but yielded the fewest connections (10 and 13%).

### In-Depth Exploration of Service Discussions

**Fig. 3:**
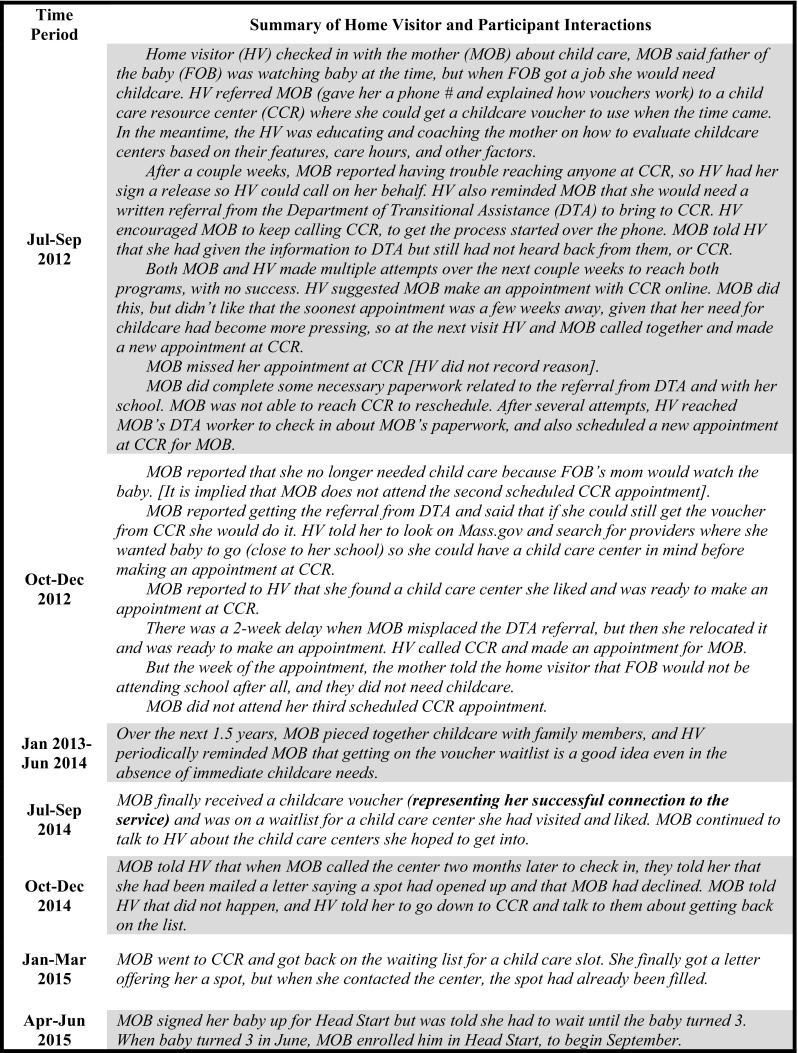
Service discussion example 1: childcare voucher

Our primary interest for the in-depth analysis of 20 participants was to generate an understanding of the pathways from referral to connection. While a presentation of findings from this study component is beyond the scope of this paper, we present two of the qualitative case studies, illustrative of HV-participant interactions around service coordination, and highlighting the range and complexity of the HVs’ roles vis a vis their clients. As background, the participant in the first example (Fig. 3) was enrolled in the program for 2.94 years, during which she received 64 home visits. She had a total of 61 service discussions with her HV,  which included eight referrals, and eight connections, across four service areas. In this example the HV and participant talked about the service—a childcare voucher program—for more than 2 years before the participant made a connection. The HV provided instrumental support (e.g., contacting agencies and scheduling appointments on the participant’s behalf,); encouragement to continue pursuing the service, persistent checking-in, which seemed to keep the goal of service connection on the participant’s radar; and reminders to complete tasks necessary to advance her goal. The HV continued to work with the young woman on accessing child care through multiple setbacks, including missed appointments, administrative complications, and frequent mind-changing on the part of the participant.

**Fig. 4:**
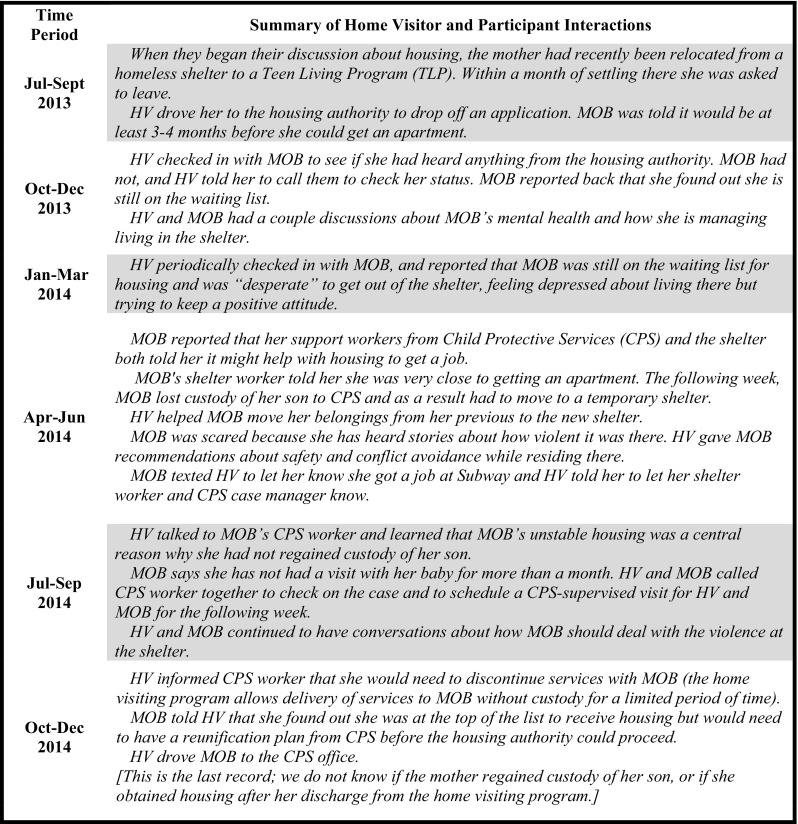
Service discussion example 2: subsidized housing

The second example (Fig. 4)—a discussion about housing services that lasted more than a year—did not end in a connection to the desired service (subsidized apartment). This participant was enrolled in the home visiting program for 2.23 years, had 46 home visits, and had 48 service discussions. Of the thirteen referrals this participant received, across nine service areas, nine resulted in a connection. This discussion underscores the diversity of ways in which HVs support young mothers; in this example, the HV did not make any referrals, and did not play a connecting role, deferring instead to others to arrange the participant’s housing placements. Rather, the HV focused on supporting the participant as she dealt with multiple housing transitions, providing instrumental and emotional support such as helping the young woman move her belongings, and offering advice to improve the quality of her living arrangement experiences. While the HV did not help the participant to secure stable, long-term housing during her tenure in the program, she remained a consistent support while this young mother navigated what was clearly an extremely challenging time in her life.

## Discussion

Results of this study suggest that the processes HVs engage in to connect participants to services are often complex, following a long and circuitous path with myriad steps involved (including those that involve trying to repair or resolve past challenges), multiple parties participating in the process, and fairly low success rates. Key conclusions are discussed below, followed by research limitations and implications for the home visiting field.

### Look Beyond Referrals to Understand HV Effort

The few home visiting evaluations examining service coordination typically have focused on whether referrals were made (Anisfeld et al. 2004) and/or whether families were connected to services (Dodge et al. 2014; Duggan et al. 2004; Jacobs et al. 2015; Love et al. 2002; Olds et al. 1986; Silovsky et al. 2011; Williams et al. 2017). Our data, however, suggest that there are many HV behaviors beyond referrals that help participants gain access to, and navigate, local systems of care. Furthermore, as was illustrated in the housing case study, there is a wide range of outcomes—other than a connection to a service—that may result from these activities, such as an increased ability to problem-solve or persist in the face of challenges. The home visiting field, and those to whom the field is accountable, would be well-served by a broader understanding of how home visiting programs engage in service coordination; simply counting “referrals” sheds insufficient light on the role HVs play in helping participants navigate community systems.

### HV Effort Makes a Difference

Moderate and advanced supports, such as instrumental support, encouragement, suggestions, and advice, interagency case review, and information provision were essential tools used by HVs to help participants realize connections to services. Through these supports, HVs helped participants navigate complex service requirements, encouraged tenacity in the face of failure and adversity, provided concrete supports that facilitated the application process, and reminded participants of important deadlines or appointments. Even in cases when HVs’ support did not lead to successful service connection, it helped participants endure the challenges of applying for services, and learn to advocate for themselves. Qualitative findings from this study suggest the central role HVs can play in navigating structural barriers to service access (e.g., changing eligibility requirements, lack of transportation), but they also highlight the role HVs play in ameliorating some of the emotional barriers (e.g., participants’ feelings of being disrespected, overwhelmed, defeated) families often experience during their encounters with the service system (Harris et al. 2016).

### The Majority of Referrals Do Not End in Service Connection

On average, only about 20% of HV referrals resulted in a connection. This seems low; although it is important to note that we do not have an empirical frame of reference for this finding. The qualitative analyses illuminated the multiple complications that tended to arise during the pathway from referral to connection (or lack thereof). The amount of HV effort required to connect participants to services varied by service area; it took considerably more intensive HV effort to connect participants with housing and early education, for instance, than to maternal health and food/nutrition services. These findings map neatly onto the program and policy landscape of in which MA MIECHV was operating at the time. For instance, MA was ranked near the top of the nation in terms of coverage by health insurance (The Commonwealth Fund 2017), Women, Infants, and Children Food and Nutrition Service (WIC) (U.S. Department of Agriculture, January 2015), and Supplemental Nutrition Assistance Program (SNAP) (U.S. Department of Agriculture, February 2014), and near the bottom for housing (National Low Income Housing Coalition 2016) and child care affordability (Economic Policy Institute 2016). Findings from this study are further confirmation that linking participants to needed services is contingent on the quality, capacity, and strength of the service systems to which they are being referred.

### Limitations

This study, a first attempt at understanding a complicated aspect of home visiting service delivery, had several limitations. First, due to our focus on service-related activities, we do not know what proportion of all HV activities is dedicated to service coordination. While it may be assumed that these activities take time away from other activities (e.g., parenting education) that home visiting programs believe to be important, this is not clear from the data. Future research should examine how service coordination fits into HVs’ workloads, how service coordination is prioritized by home visiting programs compared to other goals, and the relative benefits to participants resulting from HVs’ focus on service coordination versus other program goals. Second, there are limitations associated with using program records as the sole data source; HVs vary widely in their ability to consistently and thoroughly document services in the MIS, and without another primary data source with which to cross-reference the data, there was no way to verify their accuracy. Future studies should include data collected from participants as well, allowing for additional perspectives and better data triangulation. Third, the inclusion of only two program models in our study precluded analyses of differential approaches to service coordination by model; future research should examine model-specific variations in referral-related processes and outcomes. Finally, the outcome in this study was service connection, and how it was influenced by HV support activities and contextual factors related to service capacity and events in the participants’ lives. More research is needed to understand whether HVs’ efforts in this area lead to improvements in families’ well-being.

### Implications and Conclusions

Writing about home visiting more than two decades ago, Weiss noted that “the visitor intent on providing a holistic and family-focused service often uncovers family needs beyond those related narrowly to parenting practices or whatever the single primary focus of the program might be…” (Weiss 1993). With the advent of MIECHV, and its requirement that HVs regularly screen and refer participants in areas such as mental health, substance abuse, and domestic violence, HVs are increasingly likely to uncover needs for which there simply are not available community services—a reality that is challenging for both the provider and the family (Garg et al. 2016). Findings from this study highlight the lack of concrete resources for families in need, and the consequent difficulty experienced by HVs attempting to connect these families to services; housing in particular has emerged as a driving unmet need.

It is clear that the young women participating in home visiting programs are in profound need of access to a community system of care, and that HVs play a crucial role in helping these participants navigate this system. On the one hand, HVs are well-positioned to do this kind of work; on the other hand, service coordination is not the primary focus of home visiting; in fact, service coordination is currently seen as marginal to most home visiting services in MA, and is not part of the core training curriculum. And while we do not know if, say, time spent helping a participant complete a housing application detracts from a HV’s ability to teach about developmental milestones, there is research suggesting that HVs could better perform the essential tasks of home visiting, and families would be better poised to benefit from the services, if families had adequate resources in place (Folger et al. 2016; Lowell et al. 2011; McBride and Peterson 1997; Weiss 1993). One solution that has been adopted with demonstrable success in several home visiting models (Ayton and Joss 2016; Folger et al. 2016; Love et al. 2002; Lowell et al. 2011) is to have a more robust service coordination strategy in place at the program level, perhaps in the form of a dedicated case manager/service coordinator.

Findings from this study confirm the inextricability with which HVs are embedded in community systems of care. They not only are providing essential direct services to young mothers, but also are working behind the scenes as conduits between participants and this system, facilitating access to services by informing participants about the existence and functions of the services, interpreting complicated policies, imparting skills that can be used to pursue needed services in the future, and providing emotional support throughout. Perhaps the true capacity of HVs to influence family outcomes is yet to be discovered as a result of the burden that service coordination represents. Perhaps, also, once the laborious efforts of these frontline staff are more fully understood, it will be easier to identify sustainable program and policy solutions to the challenges of service coordination within the home visiting context.
