# Correction to: Getting to the Warm Hand-Off: A Study of Home Visitor Referral Activities

**DOI:** 10.1007/s10995-018-2618-7

**Published:** 2018-08-22

**Authors:** Jessica Goldberg, Jessica Greenstone Winestone, Rebecca Fauth, Melissa Colón, Maria Verónica Mingo

**Affiliations:** Tufts University, 574 Boston Ave., Medford, MA 02155 USA

## Correction to: Maternal and Child Health Journal 10.1007/s10995-018-2529-7

The article “Getting to the Warm Hand-Off: A Study of Home Visitor Referral Activities”, written by Jessica Goldberg, Jessica Greenstone Winestone, Rebecca Fauth, Melissa Colón and Maria Verónica Mingo, was originally published electronically on the publisher’s internet portal (currently SpringerLink) on 02 June 2018 without open access. With the author(s)’ decision to opt for Open Choice the copyright of the article changed on 17 July 2018 to © The Author(s) 2018 and the article is forthwith distributed under the terms of the Creative Commons Attribution 4.0 International License (http://creativecommons.org/licenses/by/4.0/), which permits use, duplication, adaptation, distribution and reproduction in any medium or format, as long as you give appropriate credit to the original author(s) and the source, provide a link to the Creative Commons license and indicate if changes were made.

The original article has been corrected.

